# Long-term, non-invasive FTIR detection of low-dose ionizing radiation exposure

**DOI:** 10.1038/s41598-024-56491-7

**Published:** 2024-03-13

**Authors:** Jamie L. Inman, Yulun Wu, Liang Chen, Ella Brydon, Dhruba Ghosh, Kenneth H. Wan, Jared De Chant, Lieselotte Obst-Huebl, Kei Nakamura, Corie Y. Ralston, Susan E. Celniker, Jian-Hua Mao, Peter H. Zwart, Hoi-Ying N. Holman, Hang Chang, James B. Brown, Antoine M. Snijders

**Affiliations:** 1 Biological Systems and Engineering Division, Lawrence Berkeley National Laboratory, 1 Cyclotron Rd, Berkeley, CA 94720 USA; 2 Environmental Genomics and Systems Biology Division, Lawrence Berkeley National Laboratory, 1 Cyclotron Rd, Berkeley, CA 94720 USA; 3 Department of Statistics, University of California, Berkeley, CA 94720 USA; 4 Molecular Biophysics and Integrated Bioimaging Division, Lawrence Berkeley National Laboratory, 1 Cyclotron Rd, Berkeley, CA 94720 USA; 5 Accelerator Technology and Applied Physics Division, Lawrence Berkeley National Laboratory, 1 Cyclotron Rd, Berkeley, CA 94720 USA; 6 Present Address: Paul G. Allen School of Computer Science and Engineering, University of Washington, Seattle, USA

**Keywords:** Biological techniques, Biophysical methods, Optical spectroscopy, Diagnostic markers

## Abstract

Non-invasive methods of detecting radiation exposure show promise to improve upon current approaches to biological dosimetry in ease, speed, and accuracy. Here we developed a pipeline that employs Fourier transform infrared (FTIR) spectroscopy in the mid-infrared spectrum to identify a signature of low dose ionizing radiation exposure in mouse ear pinnae over time. Mice exposed to 0.1 to 2 Gy total body irradiation were repeatedly measured by FTIR at the *stratum corneum* of the ear pinnae. We found significant discriminative power for all doses and time-points out to 90 days after exposure. Classification accuracy was maximized when testing 14 days after exposure (specificity > 0.9 with a sensitivity threshold of 0.9) and dropped by roughly 30% sensitivity at 90 days. Infrared frequencies point towards biological changes in DNA conformation, lipid oxidation and accumulation and shifts in protein secondary structure. Since only hundreds of samples were used to learn the highly discriminative signature, developing human-relevant diagnostic capabilities is likely feasible and this non-invasive procedure points toward rapid, non-invasive, and reagent-free biodosimetry applications at population scales.

## Introduction

Biological indicators of human exposure to ionizing radiation are pertinent to screen large numbers of people potentially exposed to radiation, e.g. after a nuclear accident or a radiological attack. The current gold standard in radiation biodosimetry uses cytogenetic measurements of chromosomal abnormalities in metaphases of cultured lymphocytes^1–3^. This method has played an important role in estimating radiation dose in atomic-bomb survivors in Hiroshima and Nagasaki, Japan^4^, individuals exposed after the Chernobyl nuclear^5–7^, and in the Fukushima Daiichi accident^2,8,9^. Biochemical biomarkers of radiation exposure have been developed as an alternative or supplementary method for detecting radiation exposure^10^. They include transcriptomic and proteomic biomarkers that are activated as part of the damage response pathways after radiation exposure including DNA repair, cell cycle functions and apoptosis^11^. However, there are several limitations including the relatively long processing time of cytogenetic based dosimetry (from days to one week), the need for invasive blood collections and the limited sensitivity in the low dose radiation range (< 1 Gy). To facilitate epidemiological studies, alternative approaches are needed that non-invasively detect radiation exposures at doses well below 1 Gy and at time points that are weeks to months after radiation exposure.

A main challenge of risk assessment for persons potentially exposed to ionizing radiation is in measuring the biochemical changes non-invasively and in real-time. To this end, we chose skin as our target as it is readily accessible in a way that blood and internal organs are not. The cutaneous radiation response to high doses of radiation consists of a characteristic temporal pattern depending on radiation quality, absorbed dose, dose rate and individual sensitivity. Clinical acute reactions include erythema, changes in pigment levels and depilation followed by desquamation and ulceration at extremely high doses. At the molecular level, the production of cytokines immediately after radiation induced tissue damage mediates cell–cell communications and activates repair mechanisms including late-stage fibrosis. The immediate cytokine response patterns (hours to days after exposure) have been used as radiation biomarkers to estimate dose and time after exposure. Our study examines in live mouse ear pinnae low dose radiation where no gross acute reactions are detectable nor is fibrosis observed over time. Thus, more sensitive detection methods are required for non-invasive real-time detection.

It has been established that damage to biomolecules proteins, lipids or nucleic acids and formation of reactive small molecules are reliable reporters for ionizing radiation induced effects^12–16^. Various techniques have been used to detect these damages, such as but not limited to chemiluminescence-based HPLC or GC-mass spectrometry for detection of lipid peroxidation^17–20^, GC-mass spectrometry or enzyme-linked immunosorbent assay (ELISA) for detecting DNA damages^21,22^, flow cytometry for detecting DNA damages^23,24^ or cell cycle perturbations^25,26^, label or label-free mass spectrometry for profiling protein modifications^27–30^. Many studies have demonstrated the capability of Raman spectroscopy and Fourier transform infrared (FTIR) spectroscopy to detect changes in DNA, lipid, protein and carbohydrates in exposed samples^31–39^. Long term radiotherapeutic changes in plasma have been shown by FTIR on patient-derived samples^40^. In addition, a number of non-invasive spectroscopy-based methods for radiation biodosimetry purposes have been explored. For example, in vivo electron paramagnetic resonance biodosimetry has been used for biodosimetry in nail^41,42^, hair^43^ and teeth^44^. Raman and photoluminescence (PL) spectroscopy were used to study radiation effects on human hair samples^45^. Here we use the FTIR spectroscopy imaging approach to non-invasively detect and track simultaneously changes in the composition and molecular structure of the various cellular components in the ear pinnae of living mice post exposure.

FTIR spectroscopy is one of the most sensitive analytical tools for detecting changes in composition and molecular structure in biological systems. Many common biomolecules such as proteins, lipids, carbohydrates, and nucleic acids as well as metabolites have distinct infrared-active vibrational modes that produce fingerprint-like absorption spectral features in the mid-infrared region^46,47^. These spectral features are specific to the functional groups in the molecule and therefore their composition and structure. In the last 20 years, a number of studies have demonstrated the potential of FTIR imaging as a diagnostic tool for the analysis of disease states of cells and tissues^48–57^ and for detecting radiation-induced changes in various cellular components^15,32,58–60^. FTIR can be performed in transmission, reflection (transflection), or attenuated total reflection (ATR) configurations. The millimeters-thick skin of the mouse ear pinnae attenuated most of the incident infrared light for transmission measurements while not sufficiently reflective for reflection measurements. We selected the versatile FTIR-ATR configuration in which the sampling path length is independent of the sample thickness.

We describe novel FTIR-ATR biomarkers which, when incorporated into a statistical machine learning model, can detect exposures to X-ray radiation down to 0.1 Gy at 90 days post exposure. Importantly, only hundreds of measurements on mice were needed to train this model, indicating the feasibility of developing analogs for use in human populations.

## Materials and methods

### Animal preparation

C57BL6/J and BALB/cJ mice were obtained from Jackson Laboratories and acclimated at Lawrence Berkeley National Laboratory (LBNL) for 2 weeks prior to total body irradiation (TBI) X-ray treatment at 9–12 weeks of age. Mice were housed on a 12 h light–dark cycle in standard micro-isolator cages on hardwood chips (Sani Chips; P.J. Murphy Forest Products) with enrichment consisting of crinkle cut naturalistic paper strands. Mice were maintained on ad libitum PicoLab Rodent Diet 20 (5053) and water supply with environmental humidity of 54.3% (± 10%) and temperature of 21.9 °C (± 2%). All mice were negative for all of the following pathogens: MHV, Sendai, PVM, M. pulmonis, TMEV(GDVII), Reo-3, Parvo, EDIM, LCM and Ectromelia. The study was carried out in strict accordance with the Guide for the Care and Use of Laboratory Animals of the National Institutes of Health. The Animal Welfare and Research Committee at LBNL approved the animal use protocol and this manuscript complies with the ARRIVE guidelines 2.0. Our study cohort consisted of 107 mice including 69 C57BL/6 J and 38 BALB/cJ mice (Table S1). A total of 505 spectral maps were acquired at different dosages (0, 0.1, 0.5, 1, and 2 Gy) and days (5, 14, 21, 49 and 90 days) (Table S2). Mice within a cage were identified by ear punches to the contralateral (right) ear. Mice were randomly assigned to exposure groups and no mice were excluded from the study. Mice were exposed to X-ray radiation or sham, using a Precision X-ray Inc XRAD320 320 kVp X-ray machine, operated at 300 kVp, 2 mA (dose rate of 196 mGy/min). Mice were contained in a plastic holder and irradiated on a rotating stage. Dosimetry was performed using a RadCal ion chamber (Radcal 10X6-0.18). The transverse dose profile was measured using EBT3 radiochromic films with the collimator fully open and showed that within the radius of 110 mm, the dose profile was uniform within 4% (standard deviation) across the field (Fig. S1).

### Hyperspectral infrared imaging of animals

**Figure 1:**
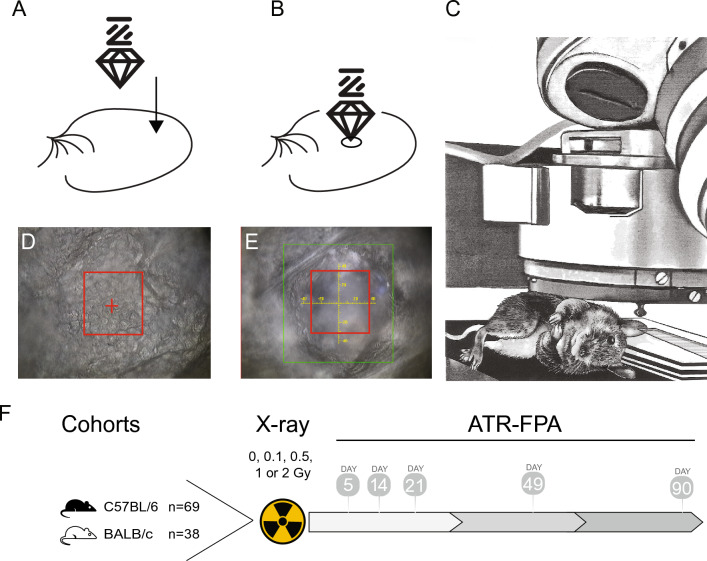
Experimental overview. (**A**) Diagram of immobilized mouse ear with germanium crystal above it. (**B**) Diagram of crystal during measurement. (**C**) Sketch of an animal under the microscope. (**D**) Phase contrast image of outermost layer of *stratum corneum* prior to measurement. (**E**) Phase contrast image of deformation of the *stratum corneum* caused by germanium crystal after measurement. (**F**) Two strains of mice C57BL/6J (n = 69) and BALB/cJ (n = 38) totaling 107 mice were generated for the study. All mice were treated with total body irradiation (TBI) of either 0, 0.1, 0.5, 1 or 2 Gy at 9–12 weeks of age. At 5-, 14-, 21-, 49- and 90-days post TBI, mice were anesthetized, and measurements of ear pinnae were collected with our FTIR-ATR-FPA system.

An overview of the FTIR-ATR hyperspectral (HS) imaging setup is shown in Fig. 1. A Hyperion FTIR microscope with a linear x–y–z translation stage equipped with a VERTEX 70 V spectrometer, a 128 × 128 pixel focal plane array (FPA) detector and stabilized visible and infrared lighting was used for hyperspectral image acquisition. Mice were anesthetized with ketamine (100–125 mg/kg) and xylazine (10–12.5 mg/kg) and the left ear pinnae was immobilized on a stack of glass slides on the x–y–z translation stage using micropore tape (3 M). The ear was positioned under the ATR objective on the stack of microscope slides and immobilized using micropore tape. A flat portion of the ear with cuboidal epithelium in the center region of the ear was identified for measurement (Fig. 1D). Care was taken to avoid vasculature and uneven surfaces. An optical bright-field image was captured prior to lifting the stage for the ear sample to make contact with the germanium crystal to collect FTIR-ATR-FPA spectra. After each measurement, a picture of the ear deformation by the crystal was captured (Fig. 1E) and the mice were returned to their cages and monitored as they recovered from anesthesia.

OPUS/ATR Software package was used to control the x–y–z sample stage and the FPA detector for automatic registration of ATR spectra. All measurements were conducted using the Bruker Opus 8.7 SP2 software package. HS infrared image data were recorded in ATR mode using the liquid nitrogen cooled 128 × 128 FPA detector and a 20 × ATR objective with a numerical aperture of 0.6. Each image consisted of 16,384 spectra. Each spectrum corresponded to the co-addition of 128 interferograms at a nominal resolution of 8 cm^–1^ with 4096 data points over the infrared spectral range of 3900–900 cm^–1^. By combining the 20 × ATR objective with the 128 × 128 FPA detector, the area of image was found to be ~ 64 × 64 um^2^, yielding a pixel size of ~ 0.5 um, consistent with those reported in literature^61^.

To measure non-invasively the skin of mice response to radiation post treatment, we calibrated a FTIR-ATR monitoring setup with a germanium crystal objective for ATR measurements coupled to a Focal Plane Array (FPA) detector. During the FTIR-ATR measurements, the ear skin of each live mouse was in direct contact with the germanium ATR element in the objective (Fig. 1A–E). The germanium has a higher refractive index (n_1_ = 4.1) than skin (n_2_ = 1.5–1.7). When the infrared light with a particular incident angle traveled through the germanium of high refractive index to the skin of low refractive index, almost all the infrared light was reflected back into the germanium, except for a small amount of the infrared light that escaped the germanium crystal and penetrated the sample as an evanescent wave with a small penetration distance beyond the crystal and was absorbed by skin. For our mid-infrared germanium element experimental setup, the wavelength-dependent penetration depth d_p_ of the evanecent wave where the wave amplitude has decreased to 1/e (i.e. ~ 37%) of its maximum value was ~ 0.2–0.65 μm. Since the light penetrates beyond the d_p_, the spectral information recorded were expected to be two to three times deeper^62,63^. The absorbance was translated into the FTIR spectrum of skin at each pixel of the FPA detector, and each spectrum was recorded as an independent, spatially resolved FTIR spectrum.

### Hyperspectral infrared image data quality control

We developed an FTIR-ATR data preprocessing pipeline on a set of 3D infrared spectral maps with fixed spatial dimension (128 × 128 pixel). We removed pixels with low quality spectra from the analysis. Note that poor quality spectra can emerge from a variety of sources, such as partial occlusion of the image by water vapor or droplets. Pixels in a given pixel pool (a segment of the image, or a collection of pixels acquired via clustering analysis) were removed if the ratio of amide signal-to-noise was below 200 and/or the ratio of the amide signal and the CO_2_ signal between 2300 and 2400 cm^–1^ were below 15^48^. For each image where more than 100 pixels were selected after this procedure, we further ranked the selected pixels by the spectra variations in the noise signature range (the lower the better) and preserved only the top 100 pixels. The remaining pixels composed the dataset for downstream analysis.

### PacMap outlier removal

To identify potential outliers, we made use of the PacMap algorithm^64^ to embed pixels in a low-dimensional space. We made use of the 1500 to 1700 cm^−1^ range, which contains the most dominant spectral bands in our classification analyses. A 2D scatter plot of the resulting embedding was created, revealing a well-defined manifold where over 99% of the pixels were located (Fig. S2). Consequently, an accepted/rejected mask was generated for each ATR image, facilitating further downstream analysis without the interference of outliers. A cursory analysis of these accepted/rejected masks did not indicate any specific spatial patterns on the detector where outliers were present, indicating random technical variation. The PacMap mask performed on par a simple selection of the central portions of images.

### Classification and statistical analysis

The hyperspectral image dataset was split into a training set consisting of 290 samples: 68 control (0 Gy) and 222 treated (0.1, 0.5, 1, 2 Gy), and an independent testing set consisting of 215 samples: 49 control and 166 treated (Table S2). Our classification models were established at the pixel level on all training samples and evaluated at the pixel and sample level in the testing set.

The classification methods used in this study include classical linear or kernel-based linear algorithms – logistic regression and support vector machines, and deep neural networks. Classical machine learning algorithms were implemented with scikit-learn (version 1.0.2) and deep learning algorithms were implemented with PyTorch (version 1.13.0). For logistic regression, two different regularizations are employed, where L2 regularization benefits efficiency and L1 regularization grants better interpretability. For deep neural networks, we use 1D-CNN as our primary approach and fully-connected feedforward neural network as a contrast method. At pixel level, prior study has proposed using 1D-CNN in different domains^65–68^, which generally outperform classical machine learning methods, due to the fact that a more abundant training set can be established at pixel level allowing higher parameter depth and more sophisticated modeling techniques. For the same reason, we found out using principal component analysis was not necessary as models did not overfit on training data without dimensional reduction. Evidently, the penalization coefficient that produced the best result on testing set for L1 regularization zeroes out only 1 input feature. However, simpler linear approaches such as LASSO logistic regression provide better interpretability since each model parameter is directly linked to one hyperspectral feature. Furthermore, LASSO has more theoretical guarantees such as almost-sure uniqueness given continuously-distributed features^69^ and sign consistency even if solutions are not unique. Hence, we used LASSO logistic regression to select wavenumbers that possess high signals with respect to radiation.

## Results and discussion

### Murine skin FTIR-ATR spectroscopic image collection after ionizing radiation exposure

Our cohort included two genetically different mouse strains, C57BL/6 J and BALB/cJ, to avoid developing a radiation signature that was strain specific. Measurements were performed under anesthesia and the left inner ear pinnae was positioned under the ATR-FPA objective (Fig. 1). Given that epidermal cells in the ear pinnae range in size from 10 to 30 µm, with an average diameter of ~ 15 µm, our data set included biosignatures from up to 25 cells per image.

### FTIR-ATR spectroscopic image pretreatment pipeline

**Figure 2:**
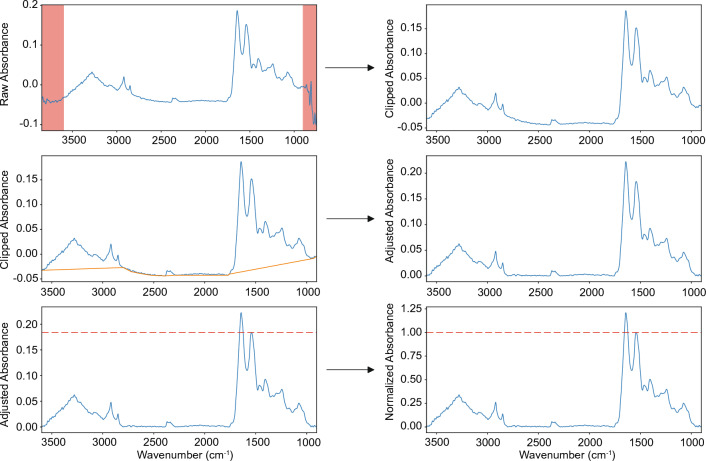
Hyperspectral image processing pipeline. Example of the procedure for quality control and normalization on hyperspectral images (range clipping, rubber-band baseline correction, peak normalization).

To reduce variance and remove redundant information, we developed a five-step procedure on hyperspectral images, including three spectra-level processing steps (range clipping, rubber-band baseline correction, peak normalization) and two pixel-level processing steps (dead pixel filling, median filter denoising). An example of the spectra-level pretreatment is shown in Fig. 2.

The pipeline included the following steps: (1) Spectra range clipping: for each pixel in the 3D map, the wavenumbers between [3600, 900] was kept to eliminate the high-variance present at the outer ends of spectral data; (2) Dead pixel filling: dead pixels, i.e. pixels in the spectral map with significantly lower signal or even no signal (usually due to instrument error), were removed using mean filtering with 5 × 5 spatial kernel; (3) Denoise: median filter with 5 × 5 spatial kernel was applied; (4) Baseline correction: baseline was established using rubber band fitting on manual control points, and then subtracted from original spectral data^48,70^; (5) Peak normalization: the baseline corrected spectral data was further normalized to the highest absorbance point in Amide II region^48^.

### FTIR peaks (wavenumbers) associated with radiation exposure

Data from the FTIR-ATR spectroscopic image processing pipeline were represented with their mean spectra from the irradiated (0.1–2 Gy) and the control (0 Gy) mice. They were transformed to second derivative spectra using the Savitzsky-Golay (SG) numerical algorithm at 3 points and 5 polynomials for more detailed analysis.

**Figure 3:**
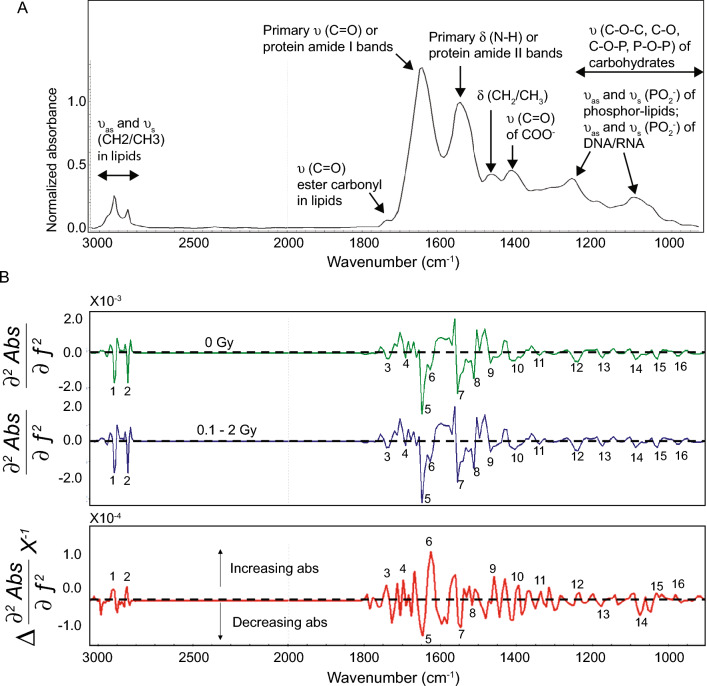
Spectral features and second derivative spectra. (**A**) Major spectral features of the murine ear skin annotated with specific biomolecules. (**B**) The mean second derivative spectra of *stratum corneum* from the control 0 Gy (green) and 0.1 to 2 Gy exposed (blue) (26,296 pixels from 274 samples) , and their difference spectrum (red). Mean spectra from the irradiated and control mouse ears in the 3050–900 cm^–1^ region were transformed to second derivative spectra (3-point Savitsky-Golay smoothing, polynomial order 5). Labeled peaks are assigned in Table 1. The difference spectrum was multiplied by − 1 for the ease of visualization purpose.

We first examined the major spectral features of the murine ear skin. An inspection of the broad features of the mean spectra from the *stratum corneum* of ears of the irradiated and control mice showed negligible changes and they are nearly superimposed (Fig. 3A). They both show typical infrared spectral absorption features of biomolecule bands in the amide I region (1700–1600 cm^−1^) and amide II region (1590–1480 cm^−1^) region, attributable to the vibrations of peptide bonds (C=O and C–N stretching, and of N–H bending modes respectively). Other major features included the symmetric and asymmetric stretching vibrations of CH_2_ and CH_3_ groups of lipids in the 3050–2840 cm^–1^ spectral region. Further overlapping bands ascribable to fatty acids and esters were detected in the 1750–1690 cm^–1^ region. Complex peaks between 1500 and 1300 cm^-1^were dominated by bending modes of > CH_2_ and > CH_3_ groups present both in amino acid side chains and in fatty acids. In the 1300–900 cm^–1^ spectral region we observed overlapping absorption peaks resulting from carbohydrates as well as phosphodiester, phosphate functional groups. The ν_as_ and ν_s_PO_2_^–^ stretching vibrations occurring at ~ 1240 cm^–1^ and at ~ 1085 cm^–1^ respectively suggested the absorption of O–P–O linkages of the polynucleotide chains in DNA and RNA. RNA showed specific absorptions at ∼1120 cm^–1^ (ribose C–O stretching), and ∼998 cm^–1^ (uracil ring stretching), while DNA exhibits peaks at ∼1020 (deoxyribose C–O stretching), and ∼964 cm^–1^ (DNA backbone motions).

We then examined the second derivative spectra in the same spectral region (Fig. 3B) and searched for specific spectral features associated with radiation exposure. Unlike the mean spectra, the impact of baseline shift (a common challenge in biological samples) on the second derivative spectrum was minimal, while the signal quality was enhanced, peak position was accurate, and the undesirable spectral features contributed by the broadband absorption constituents were eliminated. This improvement allowed us to accurately quantify the second derivative peak intensity through peak-to-peak calculation. The difference in the second derivative spectra was used for making a biochemical comparison between the irradiated and the control mice, and to infer important infrared frequencies of biomolecules which can be measured objectively (Fig. 3). Note that a characteristic feature of a second derivative spectrum is that its negative band with a minimum is at the same frequency as the maximum on the original (or zero order derivative) spectrum. The second derivative peak intensity was calculated as the intensity at the minimum. Inspection of the difference in second derivative spectrum shows notable spectral changes associated with DNA damages, lipid oxidation and accumulation, and post-translational protein modifications.

Our analysis identified sixteen best candidate biomarkers (Table 1), indicating DNA conformation changes, lipid, and protein effects. Comparative analysis of second derivative spectra revealed a small shift of the O–P–O asymmetric stretching band from ~ 1236 cm^−1^ (0 Gy) to ~ 1240 cm^−1^ (0.1–2 Gy) as the intensity increases, suggesting that the phosphate groups were less hydrogen bonded and that the DNA conformation transition from the predominant B-form to the A-DNA^32,71,72^. Local conformational changes could be associated with the damage such as the formation of pyrimidine dimers or DNA–DNA and DNA–protein cross-links^72,73^. In addition, analysis of the CH_2_ and CH_3_ stretching region (3050–2800 cm^−1^) and bending modes, and the fatty acids and ester carbonyl region (1750–1690 cm^−1^) in the second derivative spectra of the 0.1–2 Gy group revealed increases in the peak intensity of the CH_2_, CH_3_ stretching modes at 2923 cm^−1^ and at 2852 cm^−1^; CH_2_, CH_3_ bending modes at ~ 1458 cm^−1^, and ester carbonyl band at 1740 cm^−1^. However, there were no detectable shifts in the CH_2_ and CH_3_ bands. The increase in peak intensity implies lipid accumulation and increased lipid metabolism. Another important observation was oxidative damage to lipids, noted by an increase in peak intensity at 972 cm^−1^, assigned to the trans (C=C) bonds, and at 1402 cm^−1^, assigned to carbonyl groups formed during lipid oxidation^59,73,74^. This is further supported by the shift of the ester carbonyl band from 1735 to 1743 cm^−1^ in the 0.1–2 Gy group relative to the 0 Gy group. Lastly, we observed several protein effects. The second derivative spectra of the 0 Gy and the 0.1–2 Gy groups showed that a significant fraction of the *stratum corneum* protein is composed of random coils disordered secondary structure (~ 1648 cm^−1^), and to a lesser degree beta-sheet structure (1691 cm^−1^, 1626 cm^−1^^75–77^. The difference in spectrum showed that in the 0.1–2 Gy treated group of mice decreased the relative abundance of random coils disordered secondary structure in the *stratum corneum* while increasing the beta-sheet structures. The decreased intensity of amide II at ~ 1553 cm^−1^, bands assigned to tyrosine (~ 1518 cm^−1^), bands contributed by serine, threonine, and tyrosine (~ 1172 cm^−1^) and phosphorylated proteins (~ 1083 cm^−1^) suggested the sensitivity of *stratum corneum* proteins to low dose radiation.

**Table 1:** Infrared frequencies and assignment of the major infrared absorption bands in the FTIR-ATR-FPA spectra from the irradiated and control mice.

Peak number	Approximate absorption peak (cm^–1^)	Intensity (up/down), Shift (L-R)	ΔIntensity (× 10^–4^)	Assignment
1	2922		0.3	υ_as_(C–H) of saturated methylene > CH_2_ in lipids, proteins
2	2851		0.4	υ_s_(C–H) of saturated methylene > CH_2_ in lipids, proteins
3	1743		0.5	υ(C=O) of acyl groups in saturated esters like triacylglycerol
4	1691		0.2	υ(C=O) and υ(C–N) of Amide I in proteins (aide I); β-turns
5	1648		1.1	υ(C=O) and υ(C–N) of Amide I in proteins; random coils
6	1626		1.5	υ(C=O) and υ(C–N) of Amide I in proteins (aide I); β-sheets
7	1553		0.9	d(N–H) and υ(C–N) of Amide II in proteins
8	1518		0.3	Tyrosine ring stretching vibrations (–C–C/–C=C)
9	1458		0.8	δ(C–H) of CH_3_, CH_2_ in proteins, lipids
10	1402		0.6	υ(COO^–^) in proteins, lipids
11	1336		0.3	δ(C–H) of CH_2_ side chains in proteins
12	1236		0.2	υ_as_(O–P–O) of nucleic acids, phosphorylated proteins, phospholipids
13	1172		0.1	υ(–CH_2_OH) coupled with δ(–C–O–) and υ(–C–O–) of the υ(–C–OH) groups of serine, threonine, and tyrosine
14	1083		0.5	υ_s_(O–P–O) of nucleic acids, phosphorylated proteins, phospholipids
15	1024		0.1	υ(C–O) of carbohydrate side chains of proteins
16	972		0.1	trans C=C bonds

All assignments were from literature as cited in the main text.

### PacMap characterizes data manifold based on distinct spectra signatures

PacMap on spectral data ranging from 1500 to 1700 cm^–1^ produced a well-defined manifold, which categorizes the data based on the distinct forms of the Amide-I and Amide-II peak (Methods, Fig. S2). Interestingly, only a handful of observed pixels deviate from the manifold, suggesting a minimal presence of outliers and no evident partition into subclusters. There is, however, a notable contrast in the spectra distribution throughout the manifold for various doses (Fig. S2). The lack of definitive separation of subclusters within the Amide-I and Amide-II range mandates the exploration of other regions for dose differentiation, and the application of supervised learning procedures.

### Predicting ionizing radiation exposure using machine learning models

We next asked whether we can use the spectral features to distinguish irradiated mice from unirradiated mice. A variety of machine learning models of different complexity were used for the task of predicting pixel-level and sample-level radiation exposures. Ordered by complexity from lowest to highest, the models included in our analysis are: (1) Logistic Regression (LASSO, Ridge), (2) Support Vector Machines and (3) Deep Neural Networks (Fully-Connected, Convolutional). We tested these models on pixel pools filtered by two different region selection approaches – center region and PacMap region. The center region approach composed the pixel pool with pixels in the 21 × 21 center region of each image. After pretreatment and quality control, a total of 26,310 pixels from 274 samples in the training pool (Tables S3 and S4) and 18,774 pixels from 200 samples in the testing pool (Table S5) were selected for the center region. No pixels were selected from 16 training samples and 15 testing samples. These samples were removed from downstream classification analysis. The PacMAP region approach composed the pixel pool with pixels passing the PacMap outlier removal procedure. After pretreatment and quality control, 26,778 pixels from 274 samples in the training pool and 19,710 pixels from 201 samples in the testing pool were selected for the PacMap region. No pixels were selected from 16 training samples and 14 testing samples, which were excluded from downstream classification analysis.

For classification, spectra values from range [900–1800] and [2800–3050] were selected as input features. Sample-level predictions were acquired based on the majority vote of corresponding pixel-level predictions. For the center region pixel pool, all models tested in the experiments reached pixel-level and sample-level accuracies above 85% in the testing cohort, among which one-dimensional Deep Convolutional Neural Network (1D-CNN) showed the best overall classification capacity with 94.00% sample-level accuracy (Table 2). Compared to a fully-connected NN, CNN was able to resist overfitting noticeably better in our experiments. Similar to the center region, classification on the PacMap region reached pixel-level and sample-level accuracies above 85% in the testing cohort, among which 1D-CNN showed the best overall classification capacity with 92.00% sample-level accuracy (Table 2), which is calculated over 3520 unirradiated pixels from 39 samples and 15,254 irradiated pixels from 161 samples. For balanced accuracy and ROC curve, see the section below.

**Table 2:** Model comparison of radiation exposure prediction using center region and PacMapregion at the pixel- and sample-level.

Models	Center region pixels		PacMap selected pixels	
	Pixel-level	Sample-level	Pixel-level	Sample-level
LASSO-logistic regression	86.81%	87.00%	86.58%	86.83%
Ridge-logistic regression	89.20%	90.50%	88.59%	89.76%
Support vector machine	87.67%	87.00%	87.51%	87.80%
Fully connected neural network	85.25%	88.50%	85.32%	86.83%
Convolutional neural network	88.82%	94.00%	89.29%	91.22%

### Signatures exhibit flat dose–response relationships

**Figure 4:**
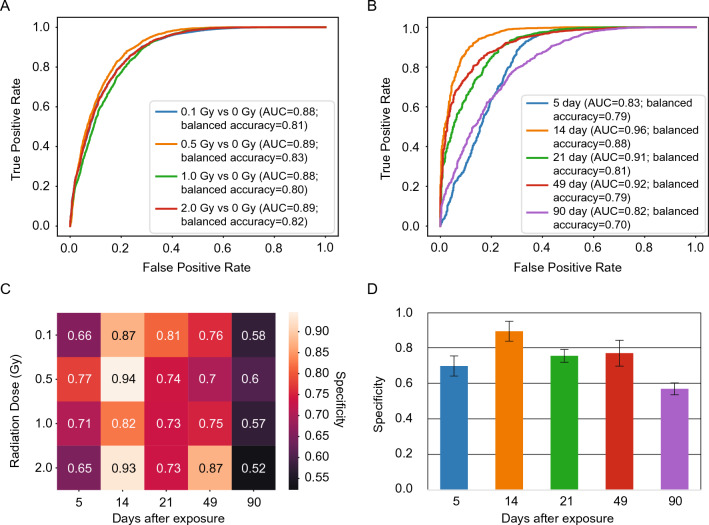
Hyperspectral image-based classification of radiation exposure. (**A**) The receiver operating characteristic (ROC) curves of predictions from center region 1D-CNN, stratified by different radiation levels. (**B**) The receiver operating characteristic (ROC) curves of predictions from center region 1D-CNN, stratified by different days after radiation exposure. (**C**) Ear ATR-FPA FTIR per-day per-radiation dose specificity with sensitivity threshold at 0.9. While specificity drops after day 21, the results are still far above random guessing (0.33 corresponds to a balanced accuracy of 0.66, p-value < 0.001). (**D**) Bar plot of specificity (at 90% sensitivity) across days after exposure. The height of each bar is the mean across doses. Error bars correspond to one SD across doses.

Interestingly, the testing accuracy did not scale with radiation dose, and the identified signatures of exposure were of similar predictive power for each radiation exposure group (Fig. 4A). We attempted to distinguish doses in a multi-class classification problem but found little power to do so with any of the models deployed (Fig. S3). Strong discriminative power for the event of radiation exposure, but not the dose in a range from 0.1 to 2 Gy is consistent with the presence of a threshold-like dose response relationship.

### Persistence of signatures of low dose ionizing radiation exposure over time

We found statistically significant discriminative power for all doses and all tested time-points out to 90 days. Our minimum balanced accuracy for the binary classification task identifying individuals exposed to any dose occurs at 90 days and at the maximum tested dose of 2 Gy (Balanced Accuracy ~ 66%, p-value < 0.001). Across all doses, classification is weakest at 5 days and 90 days, the earliest and latest time points (Fig. 4B). Detectability peaks at 14 days, where accuracy is nearly deterministic (Balanced Accuracies ~ 98%, Fig. 4C and D). We found a strong dependence on time-since-dose, detectability peaking at 14 days when cells derived from the basal stem cells of the epidermis after radiation exposure are expected to have reached the *stratum corneum*, and gradually dropping down to a balanced accuracy of ~ 75% by 90 days. The gradual drop off of detectability of our signature is likely the result of turn-over of skin epithelium and hair. However, the reduced signal at 5 days post exposure is more interesting and is consistent with a model wherein the components of our signature derive from emergent processes of host physiology, and not immediate changes in chemistry as a result of radiation exposure. This non-linear relationship with time after exposure is consistent with a model wherein a process is induced by radiation exposure, peaks somewhere around 14 days, with the system state returning closer to normal, but remaining biochemically altered and distinct out to at least 90 days.

In summary, we found that a 1D-CNN is capable of detecting signatures of low dose ionizing radiation exposure from epidermal tissues in situ for up to 90 days after exposure. We note that although 1D-CNN achieved the best classification performance on the testing sets, linear models such as logistic regression exhibited comparable performance and have advantages: the optimization process is faster and feature importance (importance of wavenumbers) can be easily acquired. These traits may be desirable to enable insights into the biochemistry captured by our classification signatures.

## Discussion

This is the first study, to our knowledge, that reports non-invasive mid-infrared biomolecular changes of radiation exposure monitored over time in living animals. We were able to track the evolving signature of the insult in three cohorts of mice providing powerful insights into biological adaptations. We used the ATR-FPA-FTIR system to record these changes in the *stratum corneum* of the mouse ear pinnae after radiation exposure. The epidermis of the ear pinnae is a stratified epithelium maintained by stem cells of the basal layer, the *stratum basale*. In mice, epidermal stem cells produce daughter cells that differentiate through the layers of the skin and end up at the surface of the *stratum corneum* in 8–10 days^78^. Thus, our method captures persistent changes in the tissue over roughly ten cycles of epidermal tissue turnover in the ear pinnae.

The FTIR-ATR spectra were recorded non-invasively at the surface of the ear pinnae and these spectra enabled us to measure how radiation exposure affected the cell composition and structures of macromolecules, such as proteins, lipids, carbohydrates and nucleic acids. Over the time we monitored the mice, the *stratum corneum* was replaced multiple times affording us insights into persistent changes arising from the stem cells of the basal layer as well as changes in the tissue microenvironment. FTIR-ATR spectrum derived from this real-time and label-free approach could be one of the most chemical information-rich and concise ways to complement the whole “-omic” of a cell, and as such, for the development of global biomarkers representing stress-and-adaptive response by different classes of biomolecules in the *stratum corneum* and epidermal stem cell populations.

We report that FTIR-ATR imaging coupled to statistical machine learning models is capable of distinguishing irradiated from control mice at doses as low as 0.1 Gy for as long as 90 days. This non-invasive procedure points toward future applications of deployable imaging devices for biodosimetry at population scales – a potentially valuable tool for radiobiology, epidemiology, and monitoring. Further, because only hundreds of samples were required to learn highly discriminative signatures, developing human-relevant diagnostic capabilities is likely feasible, e.g., generating samples from individuals undergoing radiotherapy by imaging distal (non-target) tissues subject to the low dose regime.

Of particular interest is the ever-improving interpretability of infrared measurements. As demonstrated in Table 1 and Fig. 3, the biochemistry that underlies spectral shifts upon irradiation is increasingly well-understood. Future experiments including mass spectrometry and chromatography will further enable the "inversion" of the infrared signals to map spectral peaks onto specific biochemical analytes – opening the door to non-invasive, reagent free diagnostics for a host of biomedical conditions and applications.

### Supplementary Information

Supplementary Information.

## Data Availability

Data sets and code generated during the current study are available from the corresponding authors on request. Data are located on a secure server at LBNL.
