# Supplementary material for: Long-term, non-invasive FTIR detection of low-dose ionizing radiation exposure
Source: Sci Rep. 2024 Mar 13;14:6119. doi: 10.1038/s41598-024-56491-7 (PMC10937999; doi:10.1038/s41598-024-56491-7)
Supplement: Supplementary file 1 — Supplementary Information. [file 41598_2024_56491_MOESM1_ESM.pdf]

Long-term, non-invasive FTIR detection of low-dose ionizing radiation exposure

Jamie L. Inman[1,*], Yulun Wu[2,3,*], Liang Chen[4], Ella Brydon[1], Dhruba Ghosh[4†], Kenneth H. Wan[1], Jared De Chant[5], Lieselotte Obst-Huebl[5], Kei Nakamura[5], Corie Y. Ralston[4], Susan E. Celniker[1], Jian-Hua Mao[1], Peter H. Zwart[4], Hoi-Ying N. Holman[4,#], Hang Chang[1,#], James B. Brown[2,3,#] and Antoine M. Snijders[1,#]

Supplementary Materials

Figure S1

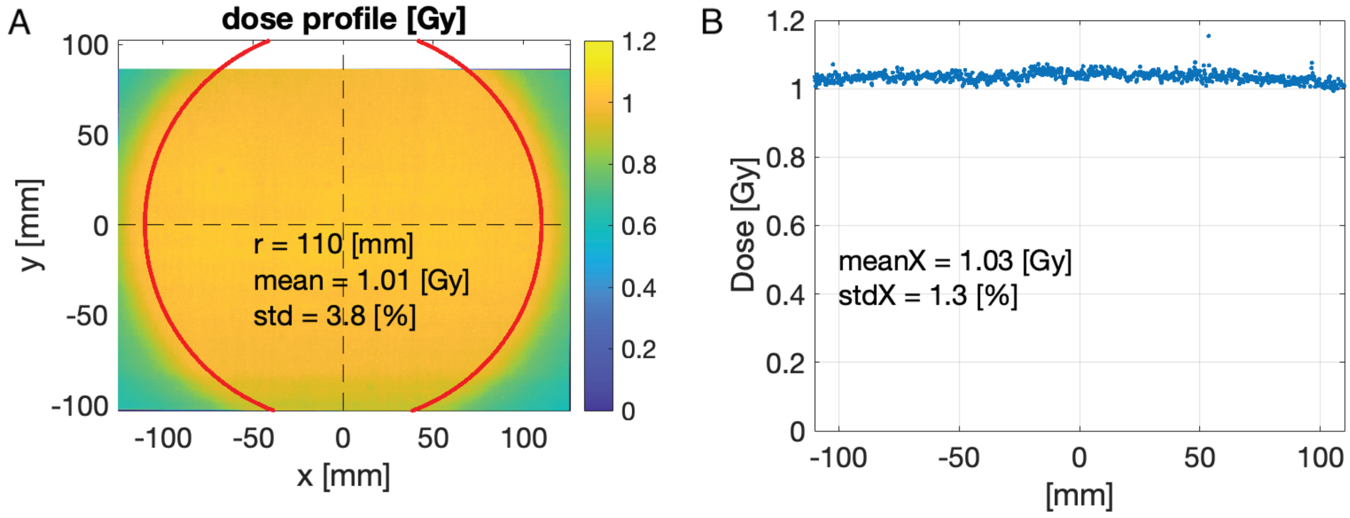

Figure S1. Dosimetry on the XRAD320: (A) Dose profile of the XRAD320 machine measured by EBT3 radiochromic film scanned at 100 dpi (254 um/pixel). The mean and standard deviation are calculated from dose values inside the red circle. (B) Horizontal lineout of dose profile. The mean and standard deviation are calculated from dose values less than 110 mm from the center.

Figure S2

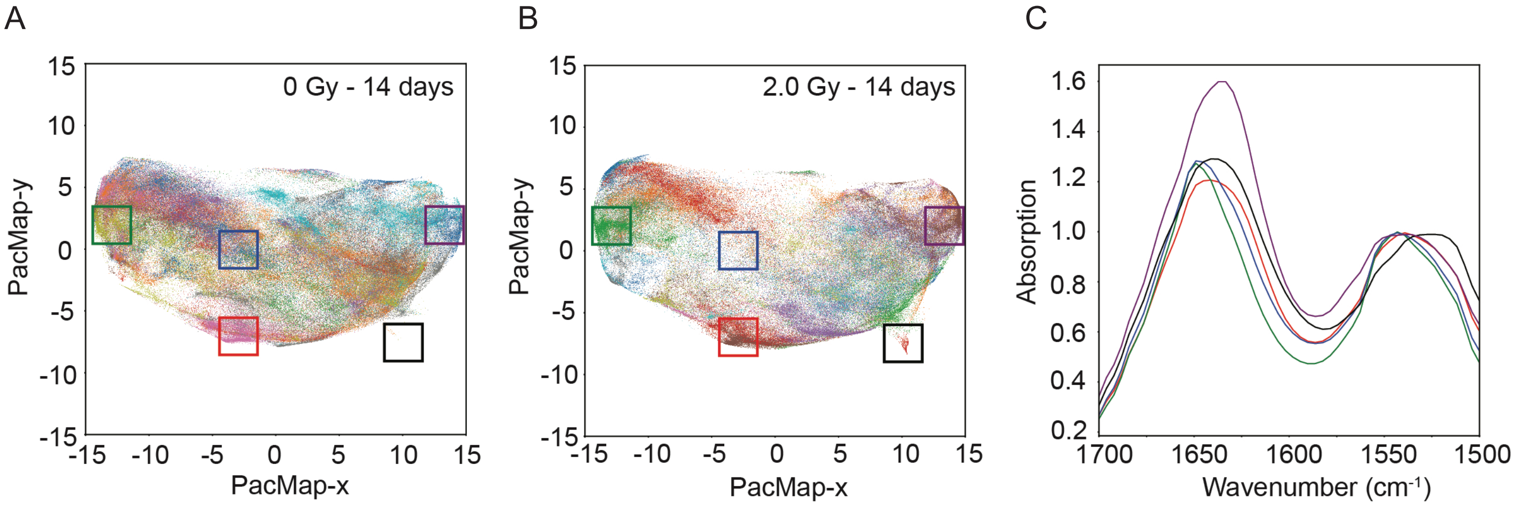

Figure S2. Dimension reduction results using PacMap. A. two-dimensional representation of control data at day 14. The scatterplot depicts all observed pixels, separated in color by the sample from which the pixels originated, within the manifold trained on all data. B. Two-dimensional representation of 2 Gy data at day 14, color coded by specimen. As can be seen, the distribution of spectra is markedly different from the control. C. Mean spectra – the mean is taken across all samples, including irradiated and control – throughout the PacMap manifold. Transversing the manifold from left to right induces an Amide-I peak widening, while a vertical movement is associated with a changing Amide-I and Amide-II peak ratio. Both these phenomena are associated with changes in secondary structure, and protein structure integrity. The color of the boxes in A and B match the color of the spectral line in C.

Figure S3

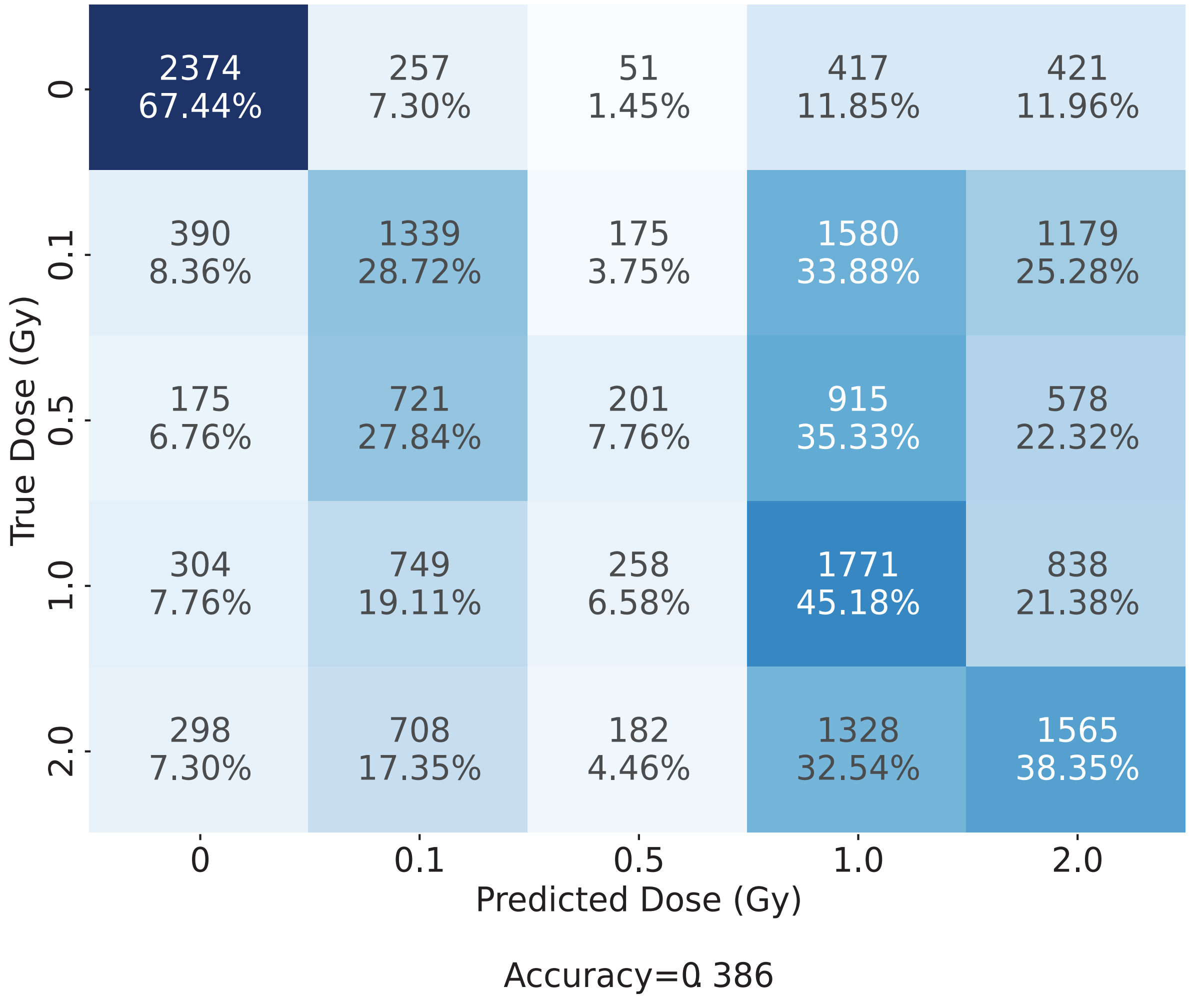

Figure S3. Confusion Matrix for the multi-class classification results for dose prediction. Color intensity is a function of % inclusion. A perfect multi-class classification result would be a diagonal matrix with 100% values along the diagonal. Off-diagonal entries quantify the degree of mis-classification. Performance is poor, but statistically different from the null of independence, where we would expect an accuracy of ~0.2 (p-value < 0.001). Hence, there is some power to distinguish dose, but only marginally. Rows have been normalized to 100%.

Table S1: Mice generated for the study by dose

Dose (Gy)	Number of mice
0	24
0.1	24
0.5	14
1.0	22
2.0	23
Total	107

Table S2: Measurements collected by time point and dose

Dose (Gy)	Total post-radiation measurements					Total
	5 days	14 days	21 days	49 days	90 days	
0	23	22	24	24	24	117
0.1	24	20	23	22	23	112
0.5	13	9	14	13	14	63
1.0	22	18	22	20	19	101
2.0	23	20	23	23	23	112
Total	105	89	106	102	103	505

Table S3: Demographics of measurements that passed quality control

Gender	Strain	Dose (Gy)	Total post-radiation measurements				
			5 days	14 days	21 days	49 days	90 days
Female	C57BL/6J	0	13	11	13	12	11
		0.1	14	10	13	12	13
		0.5	13	7	14	13	14
		1.0	14	10	14	12	13
		2.0	10	10	12	13	13
	BALB/cJ	0	3	3	2	4	5
		0.1	5	5	5	5	5
		1.0	5	5	5	5	3
		2.0	5	5	5	5	4
Male		0	3	2	2	5	5
		0.1	5	5	5	5	5
		1.0	3	3	3	3	3
		2.0	5	5	5	5	4
		TOTAL	97	81	101	99	98
		ALL	474				

Table S4: Number of measurements used for training

Gender	Strain	Dose (Gy)	Total post-radiation measurements				
			5 days	14 days	21 days	49 days	90 days
Female	C57BL/6J	0	7	6	8	7	6
		0.1	8	6	7	6	7
		0.5	7	4	8	7	8
		1.0	8	6	8	6	7
		2.0	6	6	7	7	7
	BALB/cJ	0	2	2	1	3	3
		0.1	3	3	3	3	3
		1.0	3	3	3	3	2
		2.0	3	3	3	3	3
Male		0	2	1	1	3	3
		0.1	3	3	3	3	3
		1.0	2	2	2	2	2
		2.0	3	3	3	3	2
		TOTAL	57	48	57	56	56
		ALL	274				

Table S5: Number of measurements used for testing

Gender	Strain	Dose (Gy)	Total post-radiation measurements				
			5 days	14 days	21 days	49 days	90 days
Female	C57BL/6J	0	6	5	5	5	5
		0.1	6	4	6	6	6
		0.5	6	3	6	6	6
		1.0	6	4	6	6	6
		2.0	4	4	5	6	6
	BALB/cJ	0	1	1	1	1	2
		0.1	2	2	2	2	2
		1.0	2	2	2	2	1
		2.0	2	2	2	2	1
Male		0	1	1	1	2	2
		0.1	2	2	2	2	2
		1.0	1	1	1	1	1
		2.0	2	2	2	2	2
		TOTAL	41	33	41	43	42
		ALL	200				
